# Indications, findings, and clinical impact of invasive pediatric urodynamic studies in Iraqi children

**DOI:** 10.3389/fped.2026.1885576

**Published:** 2026-08-21

**Authors:** Hawkar Khayyat

**Affiliations:** College of Medicine, Hawler Medical University, Erbil, Kurdistan Region, Iraq

**Keywords:** bladder compliance, detrusor leak point pressure, detrusor overactivity, kurdistan region of Iraq, lower urinary tract dysfunction, pediatric urodynamic study, pediatric urology, vesicoureteral reflux

## Abstract

**Background:**

Pediatric urodynamic study (UDS) provides an objective functional assessment of lower urinary tract dysfunction and assists in identifying high-risk bladder abnormalities associated with renal deterioration in children. However, regional evidence regarding pediatric UDS findings and their clinical implications remains limited in the Kurdistan Region of Iraq.

**Objective:**

To evaluate the clinical indications, urodynamic findings, and impact of pediatric UDS on patient management among children undergoing evaluation at a tertiary pediatric referral centre in Erbil, Iraq.

**Methods:**

This retrospective observational study included pediatric patients who underwent invasive UDS between January 2019 and December 2025 at a tertiary pediatric hospital in the Kurdistan Region of Iraq. Demographic characteristics, clinical indications, urodynamic findings, and treatment modifications following UDS were retrospectively collected from archived medical records and urodynamic reports. Associations between selected urodynamic abnormalities and treatment adjustment were evaluated using Fisher's exact test.

**Results:**

A total of 47 children were included, with a median age of 3.0 years (IQR, 7.0), and 78.7% were male. Vesicoureteral reflux (25.5%), posterior urethral valve (19.1%), and functional urinary incontinence (14.9%) were the most common indications for UDS. Abnormal urodynamic findings were identified in 59.6% of patients, with detrusor overactivity (21.3%), obstructive voiding (19.1%), underactive bladder (17.0%), reduced bladder compliance (19.1%), elevated detrusor leak point pressure (DLPP >40 cmH₂O; 12.8%), and abnormal sphincteric activity (10.6%) being the most frequent abnormalities. Treatment modification following UDS occurred in 48.9% of patients. Reduced bladder compliance was significantly associated with treatment adjustment (*p* = 0.001), whereas elevated DLPP and abnormal sphincteric activity were not significantly associated with management modification.

**Conclusion:**

Pediatric UDS provided clinically valuable functional information and influenced individualized management decisions in children with complex lower urinary tract disorders in the Kurdistan Region of Iraq. Reduced bladder compliance was associated with treatment modification following UDS, highlighting its clinical importance in guiding therapeutic decision-making. These findings provide one of the first regional datasets describing the indications, urodynamic findings, and management implications of pediatric UDS in Iraq.

## Introduction

Lower urinary tract dysfunction (LUTD) in children comprises a heterogeneous group of congenital, neurogenic, anatomical, and functional disorders that impair normal bladder storage and voiding function. These disorders represent an important pediatric health concern because persistent bladder dysfunction may lead to recurrent urinary tract infections (UTIs), vesicoureteral reflux (VUR), urinary incontinence, hydronephrosis, renal scarring, and progressive deterioration of upper urinary tract function (1, 2). Children with high-risk bladder dysfunction associated with elevated intravesical pressure are particularly vulnerable to irreversible renal injury, making early identification and appropriate management essential for preserving long-term renal function and improving clinical outcomes (3–5).

Although ultrasonography, voiding diaries, uroflowmetry, and post-void residual measurements are useful initial investigations, they cannot directly evaluate detrusor pressure, bladder compliance, or sphincter coordination. Consequently, invasive urodynamic studies (UDS) remain the reference standard for objectively assessing lower urinary tract function in children with suspected high-risk bladder dysfunction. Pediatric invasive urodynamic studies (UDS) provide objective assessment of bladder storage, compliance, sphincter activity, and voiding dynamics and therefore play an important role in the evaluation of children with neurogenic bladder, congenital urinary tract anomalies, refractory urinary symptoms, functional urinary incontinence, and other complex lower urinary tract disorders (6). Previous studies have demonstrated that abnormal urodynamic findings are common among pediatric urology patients and frequently influence therapeutic decision-making. Reduced bladder compliance, elevated detrusor pressure, and abnormal sphincteric activity have been associated with recurrent infections, vesicoureteral reflux, upper urinary tract deterioration, and long-term renal impairment (7, 8). Sustained detrusor pressures exceeding 40 cm H_2_O have also been linked to progressive renal damage and unfavorable urological outcomes in children with neuro-urological disorders (9).

Beyond establishing the diagnosis, invasive UDS provides clinically important information that guides individualized management, including initiation or optimisation of clean intermittent catheterization (CIC), pharmacological therapy, surgical planning, bladder augmentation, and long-term surveillance strategies (10). Nevertheless, pediatric UDS remains technically demanding and resource-intensive, particularly in low- and middle-income countries where specialized pediatric urology services, trained personnel, and advanced urodynamic equipment may be limited (11, 12). Limited access to specialized functional assessment may delay recognition of high-risk bladder dysfunction and increase the risk of preventable upper urinary tract deterioration in affected children.

Despite the burden of congenital urological anomalies, obstructive uropathies, vesicoureteral reflux, and neurogenic bladder disorders in Iraq, published evidence describing invasive pediatric urodynamic practice remains scarce. A study from Najaf reported abnormal urodynamic findings in approximately 79% of children with non-neurogenic voiding dysfunction, supporting the clinical value of pediatric UDS in lower urinary tract disorders (13). However, existing Iraqi studies have largely focused on selected clinical conditions, while comprehensive data describing the clinical indications, individual invasive urodynamic abnormalities, and subsequent management decisions among children undergoing invasive UDS are lacking. Furthermore, referral patterns, disease spectrum, availability of pediatric urology services, and access to invasive urodynamic testing may differ substantially across healthcare systems, highlighting the importance of generating locally relevant evidence. To our knowledge, few studies from Iraq, and none from the Kurdistan Region identified by our literature review, have comprehensively evaluated invasive pediatric UDS findings and their influence on clinical management (14–16).

Therefore, the present study aimed to characterize the clinical indications for invasive pediatric urodynamic evaluation, describe individual urodynamic abnormalities, and assess the influence of invasive UDS findings on subsequent clinical management among children evaluated at a tertiary pediatric referral center in the Kurdistan Region of Iraq.

## Methods

### Study design and setting

This retrospective observational study was conducted at the Pediatric Hospital in Erbil, Kurdistan Region of Iraq, a tertiary pediatric referral center providing specialized medical and surgical services for children with complex urological and congenital conditions. The center receives referrals from multiple hospitals and outpatient clinics across the Kurdistan Region for evaluation and management of lower urinary tract dysfunction, congenital urological anomalies, neurological disorders affecting bladder function, urinary incontinence, obstructive uropathies, and other complicated pediatric urological conditions. At the study center, invasive pediatric urodynamic studies (UDS) are selectively performed in children with suspected high-risk bladder dysfunction, complex congenital urological anomalies, neuro-urological disorders, or persistent lower urinary tract symptoms requiring detailed functional evaluation.

The study included all eligible consecutive pediatric patients who underwent invasive UDS between January 2019 and December 2025. Because pediatric invasive UDS is reserved for selected complex referral cases at the study center, the number of annual procedures remained relatively limited during the study period.

### Study population

The study population consisted of pediatric patients younger than 18 years who underwent invasive urodynamic assessment during the study period and had complete demographic, clinical, and urodynamic data available for retrospective review. Children referred to UDS because of suspected lower urinary tract dysfunction, congenital urinary tract anomalies, neurological disorders affecting bladder function, obstructive uropathies, functional urinary incontinence, recurrent urinary tract infections, or other complicated lower urinary tract conditions were eligible for inclusion.

Patients with incomplete medical records, missing essential urodynamic findings, or unavailable key outcome variables were excluded from the analysis. All eligible consecutive patients meeting the inclusion criteria during the study period were included to minimize selection bias. For patients who underwent multiple UDS evaluations during follow-up, only the first UDS assessment was included in the analysis to avoid duplication and preserve statistical independence. Because this was a retrospective study of all eligible consecutive patients, no formal sample size calculation was performed.

### Data collection

Data were retrospectively collected from archived medical records and urodynamic reports using a standardized data extraction form developed for the study. Extracted variables included demographic characteristics, primary clinical diagnoses, indications for UDS, previous urological interventions, clean intermittent catheterization (CIC) status, urodynamic findings, and treatment modifications following UDS evaluation.

Composite variables were created for selected clinical diagnoses to improve consistency of analysis. Neurogenic conditions included myelomeningocele, spina bifida, tethered cord, and spinal cord tumour. Obstructive uropathy included posterior urethral valve (PUV), vesicoureteric junction obstruction (VUJO), ureteropelvic junction obstruction (UPJO/PUJO), and related obstructive abnormalities. Previous major urological surgery included ureterostomy, suprapubic cystostomy, nephrostomy, and ureteric reimplantation procedures. The overall interpretation of each UDS (normal or abnormal) was based on the documented clinical assessment recorded by the treating pediatric urologist in accordance with the International Children's Continence Society (ICCS) recommendations (17).

Treatment modification was defined as any documented change in clinical management following UDS, including pharmacological therapy, clean intermittent catheterization (CIC), surgical intervention, behavioural therapy, or other treatment recommendations recorded by the treating pediatric urologist. When the specific intervention was not documented, treatment modification was recorded as present without further classification.

### Urodynamic evaluation

Pediatric urodynamic studies were performed according to institutional practice and internationally accepted pediatric urodynamic principles. UDS interpretation was conducted according to the International Children's Continence Society (ICCS) recommendations (17). The urodynamic assessments primarily consisted of filling cystometry and pressure-flow studies combined with electromyography (EMG), performed according to the clinical indication and patient cooperation.

Urodynamic evaluations were performed using age-appropriate catheters under sterile conditions. Bladder filling was performed gradually using sterile normal saline at clinically appropriate filling rates based on patient age and estimated bladder capacity. Surface electromyography electrodes were used to assess sphincter activity during bladder filling and voiding phases. Sedation was not routinely used unless clinically indicated. All urodynamic findings were interpreted by experienced pediatric urology clinicians.

Because of the retrospective study design, only variables routinely documented in the institutional electronic urodynamic database were available for analysis. Non-invasive investigations, including voiding diaries, uroflowmetry, and post-void residual volume measurements, were not consistently recorded in the database and therefore could not be evaluated in the present study. Similarly, original urodynamic tracings were unavailable for retrospective review.

The evaluated urodynamic variables included detrusor overactivity, underactive bladder, maximum cystometric capacity (MCC), obstructive voiding, bladder compliance, detrusor leak point pressure (DLPP), abnormal sphincteric activity, and the overall interpretation of the urodynamic study, as routinely documented in the institutional database. Because of the retrospective study design, the institutional database did not consistently distinguish true detrusor sphincter dyssynergia (DSD) in children with neurogenic disorders from dysfunctional voiding or other forms of abnormal sphincteric activity in non-neurogenic patients. Therefore, the broader term “abnormal sphincteric activity” is used throughout this study.

Poor bladder compliance was defined as bladder compliance <20 mL/cmH₂O (18). Detrusor leak point pressure (DLPP) was evaluated only in children in whom it could be measured during filling cystometry; consequently, DLPP was not available for all patients. When measurable, elevated DLPP was defined as >40 cmH₂O, based on established pediatric neuro-urological thresholds associated with an increased risk of upper urinary tract deterioration and renal damage (19, 20). Detrusor overactivity was defined as involuntary detrusor contractions occurring during the filling phase of cystometry. Detrusor underactivity was defined as a detrusor contraction of reduced strength and/or duration, resulting in prolonged bladder emptying and/or failure to achieve complete bladder emptying within a normal time span. Obstructive voiding was defined as increased bladder outlet resistance during voiding based on pressure-flow findings, in accordance with International Children's Continence Society (ICCS) recommendations (21).

### Statistical analysis

Data were analyzed using IBM SPSS Statistics for Windows, version 27.0 (IBM Corp., Armonk, NY, USA). Categorical variables were summarized as frequencies and percentages. Continuous variables were assessed for normality using the Shapiro–Wilk test. Normally distributed variables were presented as mean ± standard deviation (SD), whereas non-normally distributed variables were summarized as median and interquartile range (IQR).

Associations between selected urodynamic abnormalities and treatment modification following UDS were evaluated using Fisher's exact test because of the relatively small sample size and sparse subgroup frequencies. Specifically, elevated detrusor leak point pressure (DLPP), reduced bladder compliance, and detrusor sphincter dyssynergia (DSD) were assessed for their association with treatment adjustment after UDS. Analyses involving DLPP were restricted to patients in whom DLPP was measurable. Cases with missing essential urodynamic variables were excluded from the relevant analyses, and no imputation procedures were performed. All statistical tests were two-sided, and a *p*-value < 0.05 was considered statistically significant.

### Ethical considerations

Ethical approval for the study was obtained from the College of Medicine, Hawler Medical University, Erbil, Kurdistan, Iraq. The study was conducted in accordance with the principles of the Declaration of Helsinki. Patient confidentiality and privacy were strictly maintained throughout the study. Data were anonymized before analysis, and because the study was retrospective and based exclusively on review of existing medical records and archived urodynamic reports, no direct patient contact occurred during the research process.

## Results

### Baseline characteristics

A total of 47 children underwent invasive urodynamic studies during the study period. The median age was 3.0 years (IQR, 7.0), and most participants were male (78.7%). Nearly all children had a congenital urological anomaly (97.9%), while 10.6% had an underlying neurogenic condition. Obstructive uropathy was present in 29.8% of patients, 14.9% had undergone previous major urological surgery, and 10.6% were performing clean intermittent catheterization (CIC) at the time of urodynamic evaluation (Table 1).

**Table 1:** Baseline demographic and clinical characteristics of the study population (*N* = 47).

Characteristic	Value
Age, years, median (IQR)	3.0 (7.0)
Sex, *n* (%)	
Male	37 (78.7)
Female	10 (21.3)
Congenital urological anomaly, *n* (%)	
Yes	46 (97.9)
No	1 (2.1)
Underlying neurogenic condition, *n* (%)	
Yes	5 (10.6)
No	42 (89.4)
Obstructive uropathy, *n* (%)	
Yes	14 (29.8)
No	33 (70.2)
Previous major urological surgery, *n* (%)	
Yes	7 (14.9)
No	40 (85.1)
Clean intermittent catheterization (CIC) at UDS evaluation, *n* (%)	
Yes	5 (10.6)
No	42 (89.4)

### Clinical indications for invasive urodynamic study

The most common indication for invasive urodynamic study was vesicoureteral reflux (25.5%), followed by posterior urethral valve (19.1%) and functional urinary incontinence (19.1%). Less frequent indications included hypospadias, vesicoureteric junction obstruction, bladder exstrophy, epispadias, myelomeningocele, recurrent urinary tract infection, abnormal DTPA renal scan, difficult urination, spinal cord tumour, bladder diverticulum, faecal incontinence, and ureterocele (Table 2).

**Table 2:** Clinical indications for invasive pediatric urodynamic study (*N* = 47).

Clinical indication	*n* (%)
Vesicoureteral reflux (VUR)	12 (25.5)
Posterior urethral valve (PUV)	9 (19.1)
Functional urinary incontinence	7 (14.9)
Hypospadias	3 (6.4)
Vesicoureteric junction obstruction (VUJO)	3 (6.4)
Bladder exstrophy	3 (6.4)
Epispadias	2 (4.3)
Myelomeningocele (MMC)	2 (4.3)
Recurrent urinary tract infection	1 (2.1)
Abnormal DTPA renal scan	1 (2.1)
Difficult urination	1 (2.1)
Spinal cord tumour	1 (2.1)
Bladder diverticulum	1 (2.1)
Faecal incontinence	1 (2.1)
Ureterocele	1 (2.1)

### Urodynamic findings

Abnormal urodynamic findings were identified in 28 children (59.6%), whereas 19 (40.4%) had normal urodynamic studies. Detrusor overactivity was the most frequent abnormality (21.3%), followed by obstructive voiding (19.1%), underactive bladder (17.0%), reduced bladder compliance (19.1%), elevated detrusor leak point pressure (DLPP >40 cmH₂O) (12.8%), and abnormal sphincteric activity (10.6%). Elevated DLPP was measurable in 11 patients, of whom six demonstrated values exceeding 40 cmH₂O (Table 3).

**Table 3:** Urodynamic findings in children undergoing invasive urodynamic study (*N* = 47).

Urodynamic finding	*n* (%)
Detrusor overactivity	10 (21.3)
Underactive bladder	8 (17.0)
Obstructive voiding	9 (19.1)
Reduced bladder compliance (<20 mL/cmH₂O)	9 (19.1)
Elevated detrusor leak point pressure (DLPP >40 cmH₂O)^a^	6 (12.8)
Detrusor sphincter dyssynergia (DSD)	5 (10.6)
Normal urodynamic study	19 (40.4)

a DLPP was measurable in 11 patients; six had an elevated DLPP (>40 cmH₂O).

### Quantitative urodynamic parameters

The median maximum cystometric capacity was 110.0 mL (IQR, 70.0–261.0). Mean bladder compliance was 50.3 ± 28.5 mL/cmH₂O. Among the 11 children in whom DLPP could be measured, the median DLPP was 53.0 cmH₂O (IQR, 29.5–101.0) (Table 4).

**Table 4:** Quantitative urodynamic parameters.

Parameter	Value
Maximum cystometric capacity (mL), median (IQR)	110.0 (70.0–261.0)
Bladder compliance (mL/cmH_2_O), mean ± SD	50.3 ± 28.5
Detrusor leak point pressure (cmH_2_O), median (IQR)^a^	53.0 (29.5–101.0)

a DLPP was measurable in 11 of 47 children; therefore, the DLPP summary is based on *n* **=** 11.

### Clinical impact of invasive urodynamic study

Invasive urodynamic findings resulted in treatment modification in 23 children (48.9%), whereas management remained unchanged in 24 (51.1%). Documented treatment modifications included ileocystoplasty with Mitrofanoff procedure (8.5%), behavioural therapy (2.1%), intradetrusor botulinum toxin injection (2.1%), and clean intermittent catheterization with oxybutynin (2.1%). For the remaining patients with treatment modification, the specific intervention was not individually documented in the database (Table 5).

**Table 5:** Clinical impact of invasive pediatric urodynamic study on patient management (*N* **=** 47).

Clinical impact	*n* (%)
No treatment adjustment	24 (51.1)
Treatment adjustment	23 (48.9)
Ileocystoplasty with Mitrofanoff procedure	4 (8.5)
Behavioural therapy	1 (2.1)
Intradetrusor botulinum toxin injection	1 (2.1)
Clean intermittent catheterization (CIC) with oxybutynin	1 (2.1)
Treatment type not individually specified	16 (34.0)

### Association between urodynamic findings and treatment adjustment

Poor bladder compliance was significantly associated with treatment adjustment following invasive urodynamic study. All nine children with bladder compliance below 20 mL/cmH₂O underwent a change in management compared with 36.8% of those with bladder compliance ≥20 mL/cmH₂O (Fisher's exact test, *p* = 0.001). Although all children with abnormal sphincteric activity also underwent treatment modification, the association did not reach statistical significance (*p* = 0.050). Similarly, among the 11 children with measurable DLPP, treatment adjustment was not significantly associated with elevated DLPP (*p* = 0.455) (Table 6).

**Table 6:** Association between selected urodynamic findings and treatment adjustment after paediatric UDS.

Urodynamic finding	No treatment adjustment, *n* (%)	Treatment adjustment, *n* (%)	*p*-value
Elevated DLPP among patients with measurable DLPP (*n* = 11)			0.455
DLPP >40 cmH₂O	24 (63.2)	14 (36.8)	
DLPP ≤40 cmH₂O	0 (00.0)	9 (100)	
Poor bladder compliance			0.001^a^
Compliance <20 mL/cmH₂O	0 (0.0)	7 (100.0)	
Compliance ≥20 mL/cmH₂O	23 (57.5)	17 (42.5)	
Detrusor sphincter dyssynergia			0.050
DSD present	0 (0.0)	5 (100.0)	
DSD absent	23 (54.8)	19 (45.2)	

a Statistically significant at *p* < 0.05. All *p*-values were calculated using Fisher's exact test.

## Discussion

The present study evaluated the clinical indications, urodynamic findings, and management implications of pediatric urodynamic studies (UDS) performed at a tertiary pediatric referral center in the Kurdistan Region of Iraq. Approximately half (48.9%) of the study population underwent treatment modification following UDS evaluation, supporting the important clinical role of pediatric urodynamic assessment in guiding individualized therapeutic decision-making among children with complex lower urinary tract disorders.

The study population was characterized by a high prevalence of congenital and structural urinary tract abnormalities, with congenital anomalies identified in nearly all patients. Similar patterns have been reported in tertiary pediatric urology centers, where children referred for urodynamic evaluation commonly present with congenital anomalies, obstructive uropathies, neurogenic bladder dysfunction, or refractory lower urinary tract symptoms requiring specialized functional assessment (20, 22). The predominance of male patients observed in the present study is also consistent with the relatively high frequency of conditions such as posterior urethral valve (PUV) and obstructive uropathies, which occur predominantly in boys (23).

Vesicoureteral reflux (VUR) represented the most common indication for pediatric UDS in the present cohort, followed by posterior urethral valve (PUV) and functional urinary incontinence. Previous pediatric studies and international guidelines have similarly identified VUR, neurogenic bladder dysfunction, refractory lower urinary tract symptoms, functional urinary incontinence, and obstructive uropathies as major indications for pediatric urodynamic evaluation (20, 24, 25). These findings further support the role of pediatric UDS in evaluating children with potentially high-risk bladder dysfunction and complicated lower urinary tract disorders.

Abnormal urodynamic findings were identified in approximately 60% of the children, with detrusor overactivity representing the most frequent abnormality, followed by obstructive voiding, underactive bladder, reduced bladder compliance, elevated detrusor leak point pressure (DLPP), and abnormal sphincteric activity. These findings are consistent with previous pediatric studies demonstrating high rates of abnormal bladder dynamics among children referred for specialized urological assessment, particularly in tertiary referral settings where complex lower urinary tract disorders are frequently encountered (2, 8, 22). Importantly, clinical symptoms and routine imaging investigations alone may inadequately predict high-risk bladder behavior or upper urinary tract risk in pediatric patients, emphasizing the importance of objective urodynamic evaluation for accurate functional assessment, risk stratification, and individualized management planning (25, 26).

One of the most clinically important findings of the present study was the identification of reduced bladder compliance in 19.1% of patients and elevated DLPP (>40 cmH_2_O) in 6 of the 11 children in whom DLPP could be measured. Elevated intravesical storage pressure and impaired bladder compliance are recognized high-risk urodynamic parameters because they are strongly associated with vesicoureteral reflux, recurrent urinary tract infections, hydronephrosis, progressive renal deterioration, and long-term upper urinary tract damage in children with neurogenic bladder dysfunction (19, 20). Early identification of these abnormalities is clinically important because timely intervention may help reduce the risk of irreversible renal injury and long-term bladder dysfunction.

The clinical utility of pediatric UDS was further supported by the observation that treatment modification occurred in nearly half of the study population following urodynamic evaluation. Poor bladder compliance was significantly associated with treatment adjustment, whereas elevated DLPP and abnormal sphincteric activity were not statistically associated with management modification, although all children with abnormal sphincteric activity underwent treatment adjustment. These findings suggest that reduced bladder compliance may be a particularly important indicator for therapeutic intervention, consistent with previous evidence identifying impaired bladder compliance as a major risk factor for upper urinary tract deterioration (20, 27, 28). The lack of statistically significant associations for DLPP and abnormal sphincteric activity should be interpreted cautiously because only a small number of children exhibited these abnormalities, limiting statistical power.

The findings of the present study support International Children's Continence Society (ICCS) recommendations emphasizing the role of pediatric UDS in identifying high-risk bladder patterns requiring individualized management strategies (17). Importantly, the present study provides one of the first detailed regional descriptions of pediatric UDS indications, abnormalities, and management implications from the Kurdistan Region of Iraq, where published pediatric urodynamic data remain limited.

Nevertheless, several limitations should be acknowledged. First, the retrospective single-center design may limit the generalizability of the findings to other healthcare settings. Second, the relatively small sample size reduced statistical power, particularly for subgroup analyses involving uncommon urodynamic abnormalities such as elevated DLPP and abnormal sphincteric activity. Third, referral bias is likely present because the study center reserves pediatric UDS for selected complex referral cases. Fourth, some potentially important clinical variables and long-term renal outcomes were not consistently available because of the retrospective nature of the study. Fifth, potential interobserver variability in retrospective interpretation of urodynamic findings could not be completely excluded. In addition, because of the retrospective study design, the institutional urodynamic database did not consistently distinguish true detrusor sphincter dyssynergia (DSD) in children with neurogenic disorders from dysfunctional voiding or other forms of abnormal sphincteric activity in non-neurogenic patients. Consequently, abnormal sphincteric activity was analyzed as a single variable in the present study. Finally, the absence of longitudinal follow-up data limited evaluation of the long-term prognostic implications of abnormal pediatric urodynamic findings.

Despite these limitations, the findings suggest that pediatric UDS provides clinically valuable functional information that may substantially influence management decisions in children with complex lower urinary tract disorders. Reduced bladder compliance was significantly associated with treatment modification following UDS evaluation, highlighting its importance as an indicator of clinically relevant bladder dysfunction. Although elevated DLPP and abnormal sphincteric activity were not significantly associated with treatment adjustment, their established clinical relevance and the limited sample size warrant cautious interpretation of these findings. Further prospective multicenter studies with larger sample sizes are warranted to validate these findings and better define the prognostic significance of pediatric urodynamic abnormalities on long-term renal and bladder outcomes.

## Conclusion

In conclusion, pediatric urodynamic study (UDS) provided clinically valuable functional and diagnostic information for children with complex lower urinary tract disorders managed at a tertiary pediatric referral centre in the Kurdistan Region of Iraq. Nearly half of the study population underwent treatment modification following UDS evaluation, underscoring the important role of UDS in guiding individualized management strategies. Reduced bladder compliance was the only urodynamic abnormality significantly associated with treatment adjustment following UDS evaluation, highlighting its clinical importance in guiding therapeutic decision-making.

Although the findings should be interpreted with caution because of the retrospective single-centre design and relatively small sample size, this study provides important regional evidence regarding the clinical indications, urodynamic findings, and management implications of pediatric UDS. Further prospective multicentre studies with larger sample sizes and longitudinal follow-up are warranted to validate these findings and better define the prognostic significance of pediatric urodynamic abnormalities for long-term bladder and renal outcomes.

## Data Availability

The raw data supporting the conclusions of this article will be made available by the authors, without undue reservation.
